# Clot value: Why mechanical thrombectomy may be the smartest investment in iliofemoral deep vein thrombosis care

**DOI:** 10.1016/j.jvsv.2026.102536

**Published:** 2026-05-25

**Authors:** Nicolas J. Mouawad

**Affiliations:** Division of Vascular & Endovascular Surgery, McLaren Health, Bay City, MI

The frenzy of venous thrombectomy has catapulted a traditionally medically managed pathology into a very crowded interventional landscape with a myriad of devices on the market, each with its specifications, mechanisms of action, and, of course, cost. The findings of this cost-effectiveness analysis by Hafeez et al^1^ are highly significant because they address one of the most important unresolved questions in contemporary venous disease management: how does one optimize outcomes for patients with iliofemoral deep vein thrombosis (IFDVT) while maintaining responsible health care spending in an increasingly financially constrained environment? IFDVT is not simply an acute thrombotic event requiring anticoagulation; it accounts for significant annual health care spending, not just at the index event, but also for potentially lifelong consequences, namely, the development of post-thrombotic syndrome (PTS), chronic pain, edema, venous claudication, and venous ulceration.^2^^,^^3^ These long-term complications impair quality of life, decrease productivity, and create substantial downstream costs for patients and health care systems alike. Therefore, any therapy capable of decreasing the burden of PTS while remaining economically justified deserves serious attention.

The study's most significant contribution is its demonstration that mechanical venous thrombectomy (MVT) may offer the most favorable balance between clinical benefit and economic value when compared with both anticoagulation alone and catheter-directed thrombolysis (CDL). Historically, treatment decisions in IFDVT have often been framed as a binary choice between conservative anticoagulation and thrombolysis-based intervention. The emergence of modern thrombectomy platforms has changed that paradigm by allowing rapid thrombus extraction, primarily in a single session, without prolonged lytic infusion and with reduced bleeding exposure. This analysis suggests that these procedural advantages translate not only into clinical efficiency, but also into measurable gains in quality-adjusted life-years at acceptable cost thresholds.

Contemporarily, the dominance of MVT over CDL is particularly noteworthy.^4^, ^5^, ^6^, ^7^ Although CDL represented an important advance in prior decades, its limitations are well recognized: intensive care monitoring, repeat imaging procedures, longer hospital stays, and the inherent risk of major or minor bleeding associated with thrombolytic drugs. Even if CDL can improve symptoms or reduce PTS severity in selected patients, the resource utilization associated with this approach is substantial. In contrast, the single-session nature of MVT, combined with shorter hospitalization and minimal reliance on thrombolytics, appears to create a more efficient pathway of care. In an era of bundled payments, hospital cost containment, and value-based medicine, this distinction carries major practical importance. In fact, endovascular strategies for the treatment of DVT that avoided thrombolytics favored MVT with the lowest episode-of-care costs.^8^

MVT remained cost-effective across wide ranges of procedural cost, age variation, and PTS risk assumptions, and was favored in the vast majority of probabilistic simulations.^1^ That suggests that the findings are not dependent on a narrow set of optimistic assumptions, but may reflect a broadly durable economic advantage. For clinicians, this means MVT may be justified not only in ideal candidates, but also across a realistic spectrum of patients with symptomatic IFDVT. Equally important are the implications for patient-centered care. Younger, active patients with a long life expectancy stand to lose the most from chronic venous morbidity. Preventing moderate or severe PTS in these individuals may preserve mobility, work capacity, exercise tolerance, and psychosocial well-being for decades. The value of that benefit cannot be captured by procedural cost alone.

Ultimately, these findings reinforce a larger shift in vascular care: success should no longer be judged solely by technical thrombus removal or short-term patency, but by long-term quality of life and economic sustainability. Although randomized comparative trials remain necessary, this study provides compelling evidence that MVT may represent the most rational cost-effective modern treatment strategy for appropriately selected patients with IFDVT.

*The opinions or views expressed in this commentary are those of the authors and do not necessarily reflect the opinions or recommendations of the Journal of Vascular Surgery: Venous and Lymphatic Disorders or the Society for Vascular Surgery.*

## Funding

None.

## Disclosures

N.J.M. is a consultant for Boston Scientific, Inari Medical/Stryker, and Inquis Medical.
